# Multimodal profiling uncovers an aging-associated ETS1-MYO1B vascular niche responsive to ipragliflozin

**DOI:** 10.3389/fcell.2026.1968248

**Published:** 2026-09-25

**Authors:** Mingzhu Jin, Yue Zhang, Haien Wu, Bo You, Ling Li, Xiuping Han, Yan Wu

**Affiliations:** 1 Department of Dermatology, Shengjing Hospital, China Medical University, Shenyang, China; 2 Department of Pathology, Cancer Hospital of Dalian University of Technology, Liaoning Cancer Hospital & Institute, Shenyang, China; 3 Department of Dermatology, The Seventh People's Hospital of Shenyang, Shenyang, China; 4 Department of Nursing, Cancer Hospital of Dalian University of Technology, Liaoning Cancer Hospital & Institute, Shenyang, China

**Keywords:** endothelial-to-pericyte transition, ETS1, ipragliflozin, MYO1B, skin aging, vascular niche

## Abstract

**Background:**

Tissue aging involves complex changes in vascular, immune, and stromal compartments that collectively reshape the local tissue ecosystem and may contribute to age-related diseases, including cancer, fibrotic diseases, and cardiovascular disease. However, it remains unclear whether reproducible, cell-specific vascular niche states emerge during aging and whether these states can be therapeutically targeted.

**Methods:**

A machine-learning single-cell age-clock model was constructed using three published human skin single-cell transcriptomic datasets and validated against chronological age. Vascular subpopulations were resolved by NMF clustering and diffusion pseudotime analysis. These findings were further validated in 15 human skin samples using H&E-based digital image analysis and immunohistochemistry. An inflammatory HMEC-1 model was used for RT-qPCR, flow cytometry, SA-β-gal staining, siRNA knockdown, and ChIP-qPCR. Structure-based virtual screening of an FDA drug library, molecular dynamics simulation, and biolayer interferometry were used to identify and validate ETS1-targeting compounds. Efficacy was assessed *in vivo* in a D-galactose-induced aging model treated with oral ipragliflozin.

**Results:**

Predicted age correlated with chronological age, with vascular endothelial cells, lymphatic endothelial cells, and pericytes carrying the strongest aging signals. Aged skin showed a higher low-hematoxylin-area burden, reduced boundary-complexity variation, and reduced vascular-area heterogeneity. An aging-associated pericyte subcluster marked by MYO1B was identified, together with a continuous endothelial-to-pericyte transition (EndoPT). TGF-β1/IL-1β stimulation induced MYO1B- and PDGFRB-positive pericyte-like transition and senescence in HMEC-1 cells. ETS1 bound the MYO1B promoter and was associated with aging status. Ipragliflozin bound the transcription-regulatory region of ETS1, suppressed the ETS1–MYO1B axis, reduced EndoPT and senescence *in vitro*, and restored epidermal thickness while reducing MYO1B, PDGFRB, and PAI-1 in aged rat skin.

**Conclusion:**

Multimodal profiling identifies an aging-associated ETS1-MYO1B vascular niche characterized by heightened inflammation, endothelial-to-pericyte plasticity, and cellular senescence, and demonstrates that this niche can be therapeutically targeted with ipragliflozin. As these processes also contribute to the microenvironmental remodeling observed in cancer and other age-related diseases, this niche may provide broader insight into how vascular and stromal alterations shape tissue aging and disease susceptibility.

## 1 Introduction

Skin aging is a structural and cellular process. As skin aging progresses, the thinning of the epidermis happens, dermal collagen is progressively remodeled and lost, and the capacity for repair and homeostatic turnover declines (Farage et al., 2008; Rittie et al., 2015). Senescent skin cells accumulate and adopt a senescence-associated secretory phenotype (SASP) that releases pro-inflammatory cytokines and matrix-degrading enzymes, sustaining low-grade inflammation and matrix breakdown (Coppé et al., 2008; Low et al., 2021). Single-cell studies of human skin have mapped these age-related changes across cell types, characterizing changes in the abundance and state of multiple skin cell populations during aging (Zou et al., 2021). These cellular dynamics establish a multifaceted pathological landscape of skin aging, characterized by different tissue architecture, cell state, and the local inflammatory environment.

As senescence occurs concurrently across multiple cell types, resolving the specific cell-state transitions and their chronological role during aging remains challenging. Age-associated models address this by learning the relationship between molecular changes and chronological age. Built on single-cell data, such models can quantify aging-related molecular change, and capture implicit age signals for further investigation (Horvath, 2013; Buckley et al., 2023). While these aging clocks serve as powerful descriptive and prioritization tools, they do not intrinsically establish causality. Consequently, experimental validations remain essential to demonstrate whether these genes or cell states directly drive the aging process.

The dermal microvasculature constitutes a critical component of the skin microenvironment during aging. Cutaneous blood flow and microvascular density decline with age, accompanied by disorganized branching and endothelial dysfunction, all of which constrain oxygen and nutrient delivery to the skin (Bentov and Reed, 2015; Chung and Eun, 2007). Endothelial cells form the barrier of microvessels, modulating molecular exchange, inflammatory recruitment, and tissue repair, whereas pericytes wrap the endothelium and stabilize microvessels through PDGF-B/PDGFRβ signaling, contributing to vascular maturation and tissue homeostasis (von Tell et al., 2006; Armulik et al., 2011). Recent work has shown that endothelial senescence can directly promote skin aging features including epidermis thinning and impaired wound healing (Wicaksono et al., 2025). Positioned at the interface between the circulation and the dermal matrix, the microvasculature undergoes age-related changes that can trigger widespread aging cascades throughout the cutaneous microenvironment.

The central component of this microenvironmental impact is the intrinsic plasticity of endothelial cells, notably characterized by endothelial-to-mesenchymal transition (EndoMT). During EndoMT, endothelial cells downregulate classical adherence markers like CD31 and VE-cadherin to transfer to a mesenchymal status. Under the influence of TGF-β signaling, these transitioning cells begin expressing fibroblastic markers, including α-SMA and type I collagen (Ma et al., 2020; Piera-Velazquez and Jimenez, 2012). In tissue aging, this phenotypic drift serves as a critical driver of microvascular destabilization, linking endothelial dysfunction directly to the progressive fibrotic remodeling of the dermal matrix. In addition to this classical fibroblastic transition, endothelial plasticity can redirect cells toward an alternative vascular lineage via an endothelial-to-pericyte transition (EndoPT). Accumulating evidence indicates that this EndoPT is reactivated in post-injury vascular remodeling (Chen et al., 2016). However, whether this cellular transition is sustained during chronic, age-associated microenvironmental stress remains unexplored.

Current anti-aging interventions primarily focus on the elimination of senescent cells alongside SASP suppression, and the modulation of mTOR, AMPK, or NAD + supplementation (López-Otín et al., 2023; Zhang et al., 2023). In the study of skin aging, conventional anti-aging treatments primarily center on repairing the epidermal barrier, dealing with local oxidative stress, or stimulating fibroblast-driven collagen synthesis. By contrast, intervention studies targeting deeper tissue niches—particularly the dermal vascular niche, endothelial–pericyte interactions, and microvascular stability—remain limited.

In this study, we combined a single-cell skin age-clock model with local histological samples, and constructed an *in vitro* HMEC-1 inflammatory model and an *in vivo* D-galactose-induced rat skin aging model to focus specifically on aging-associated changes within the dermal vascular niche. We first constructed a machine-learning single-cell age-clock model, which subsequently enabled us to identify an aging-associated pericyte population. Within this population, we characterized a potential regulatory axis between the transcription factor ETS1 and the motor protein MYO1B, which was found to modulate cellular senescence and EndoPT. As a further intervention strategy, structure-based screening of an FDA drug library identified the SGLT2 inhibitor ipragliflozin, whose therapeutic efficacy was evaluated *in vitro* and *in vivo* to assess its anti-aging potential.

## 2 Materials and methods

### 2.1 Human skin single-cell transcriptomic datasets and computational analysis

Three published human skin single-cell transcriptomic datasets were included to analyze skin aging-related cellular niches: the human skin aging atlas reported by Zou et al. (Zou et al., 2021), the GSE130973 human skin aging dataset reported by Solé-Boldo et al. (Solé-Boldo et al., 2020), and the healthy adult human skin cell atlas reported by Reynolds et al. (Reynolds et al., 2021). These datasets were used for harmonized cell-type annotation, vascular-associated subpopulation analysis, age-related expression analysis and externally supported single-cell age-clock modeling.

Single-cell data were processed using standard workflows for cell filtering, normalization, dimensionality reduction and visualization (Hao et al., 2021; Wolf et al., 2018; Virshup et al., 2021). Batch and dataset effects were addressed using scVI (Gayoso et al., 2022). Cell types were initially annotated within the CellTypist framework and then manually curated according to annotated adult healthy skin atlases and canonical markers (Domínguez et al., 2022). Major annotated categories included vascular endothelial cells, lymphatic endothelial cells, pericytes, fibroblasts, keratinocytes, immune cells and other skin-associated populations.

To build the age-prediction model, processed data were aggregated by sample and cell type to generate pseudo-bulk expression matrices. Cell-type-specific expression features were used to train age-prediction models. Partial least-squares regression and ridge regression were implemented with standard machine-learning and statistical packages, and model performance was evaluated by cross-validation, correlation between predicted and chronological age, and calibration slope (Pedregosa et al., 2011; Seabold and Perktold, 2010). Hyperparameter optimization was performed via systematic grid search. The search space included: bagging iterations (8, 10), gene retention fraction (0.6, 0.8), number of highly variable genes per cell type (200, 250, 350), number of PCA components (1, 2), number of HVGs for supervised embedding (2000, 3000), minimum cell types (3, 4), top-k cell types (2, 3), and robust embedding options, totaling 192 parameter combinations. The optimal model was selected by minimizing a weighted loss function based on cross-validation performance. The best-performing configuration consisted of bags = 8, gfrac = 0.8, hvg = 200, kpc = 2, se_n_hvg = 3000, minct = 3, and topk = 2. Cell-type weights, model-weighted genes and prediction performance were jointly used to identify major cellular sources of aging signals. Differential expression was assessed using the Wilcoxon rank-sum test, and the Benjamini-Hochberg procedure was used to control the false-discovery rate where appropriate (Hao et al., 2021; Wolf et al., 2018).

Differential genes were further analyzed by pathway enrichment, SASP-related gene-set scoring and cell-cell communication inference. Kyoto Encyclopedia of Genes and Genomes (KEGG) pathway enrichment was performed using clusterProfiler with the KEGG database (Yu et al., 2012; Kanehisa and Goto, 2000). SASP-related gene sets were selected from SenMayo, CellAge, MSigDB/Reactome and the SASP Atlas according to the analysis (Saul et al., 2022; Tacutu et al., 2018; Basisty et al., 2020; Liberzon et al., 2015; Jassal et al., 2020). NicheNet was used to infer ligand-receptor-target regulatory relationships and to evaluate potential cell-cell communication among endothelial cells, pericytes and other vascular-niche-associated cells (Browaeys et al., 2020).

Vascular-associated cell populations were further subclustered. Vascular endothelial cells, lymphatic endothelial cells and pericytes were analyzed by consensus non-negative matrix factorization (cNMF) clustering, uniform manifold approximation and projection (UMAP) visualization, marker-gene identification and age-associated cluster-proportion analysis (Kotliar et al., 2019; Lee and Seung, 1999; McInnes et al., 2018). Endothelial and pericyte-associated state transitions were evaluated by diffusion pseudotime, and endothelial, pericyte, senescence and EndoMT/EndoPT-related genes were mapped onto pseudotime trajectories (Haghverdi et al., 2016). Correlation analyses and visualization were performed in Python/R using pandas, NumPy, SciPy, statsmodels, Matplotlib, ggplot2 and ComplexHeatmap (Seabold and Perktold, 2010; McKinney, 2010; Harris et al., 2020; Virtanen et al., 2020; Hunter, 2007; Wickham, 2016; Gu et al., 2016).

### 2.2 Human skin tissue samples

The validation cohort used discarded skin tissue obtained after dermatological surgery. Samples were used for H&E staining, immunohistochemistry and digital image analysis. This study was performed in accordance with the ethical standards of the Declaration of Helsinki and was approved by the Ethics Committee of Shengjing Hospital of China Medical University (2025PS423K).

### 2.3 Hematoxylin and eosin staining and digital image analysis of the vascular niche

Skin tissues were fixed, paraffin-embedded and stained with hematoxylin and eosin (H&E). Vascular-niche regions were manually annotated in QuPath according to tissue architecture and perivascular structures, and annotations were exported for digital image analysis (Bankhead et al., 2017). The analysis workflow summarized vascular-niche regions at the sample level based on ROI masks and extracted tissue-structure, cell-density, staining-intensity, spatial-distribution and morphological features. H&E image quantification included low-hematoxylin-area burden, boundary-complexity variation, vascular-area size heterogeneity, nuclear features and matrix texture.

### 2.4 Immunohistochemistry

Paraffin sections were processed by deparaffinization, rehydration, blocking, primary-antibody incubation, detection, counterstaining and mounting. MYO1B, p21/CDKN1A and PAI-1/SERPINE1 were used to evaluate vascular-associated MYO1B expression and senescence-related markers, and PDGFRB was used to assess pericyte-associated signals. The antibody information was listed as followed MYO1B (Proteintech, 15012-1-AP), p21/CDKN1A (Proteintech, 10355-1-AP), PAI-1/SERPINE1 (Proteintech, 66261-1-Ig), PDGFRB antibody (Proteintech, 13449-1-AP) and ETS1 (Proteintech, 12118-1-AP).

### 2.5 HMEC-1 cell culture, inflammatory/TGF-β stimulation, ipragliflozin treatment and ETS1 knockdown

HMEC-1 cells were obtained from Procell (CL-0576). Cells were cultured in MCDB131 basal medium supplemented with fetal bovine serum, EGF, hydrocortisone and glutamine at 37 °C with 5% CO2.

To establish an inflammatory/TGF-β stimulation model, HMEC-1 cells were treated with recombinant human TGF-β1 (Abclonal, RP01458) and recombinant human IL-1β (Abclonal, RP00002) at 10 ng/mL each for 7 days. Ipragliflozin (MCE, HY-14894) was used to treat HMEC-1 cells at 10 μM. ETS1 knockdown was performed by siRNA transfection. SiRNA was synthesized by Obio Technology. Cells were seeded and transfected with ETS1 siRNA or negative-control. Knockdown efficiency was verified by RT-qPCR. The ETS1 siRNA sequence was sense, ACU​UGC​UAC​CAU​CCC​GUA​C; antisense, GUA​CGG​GAU​GGU​AGC​AAG​U.

### 2.6 RNA extraction and reverse-transcription quantitative PCR

Total RNA from cells or tissue samples was extracted using the Vazyme FastPure Cell/Tissue Total RNA Isolation Kit (RC101). Reverse transcription was performed with Vazyme HiScript III RT SuperMix for qPCR (R323), and qPCR was performed with Vazyme ChamQ Universal SYBR qPCR Master Mix (Q711). Candidate qPCR primers were obtained from PrimerBank (Wang et al., 2012). Relative expression was normalized to an internal reference gene and calculated using the 2^-ΔΔCt method.

### 2.7 Flow cytometry

HMEC-1 cells were collected after the indicated treatments and processed for fixation, permeabilization and antibody incubation. PDGFRB and MYO1B signals were used to evaluate endothelial-to-pericyte-like state changes. Isotype controls were set before acquisition, and debris and aggregates were excluded.

### 2.8 SA-β-gal staining

Senescence-associated β-galactosidase staining was used to evaluate cellular senescence. Treated cells were stained using the Beyotime Senescence beta-Galactosidase Staining Kit (C0602) according to the manufacturer’s instructions.

### 2.9 Immunofluorescence staining

Cell samples were fixed, permeabilized and blocked, incubated with antibodies against PDGFRB, MYO1B and CD31, and then incubated with fluorescence-conjugated secondary antibodies. Nuclei were counterstained with DAPI. The CD31 antibody was Proteintech 32387-1-AP.

### 2.10 Chromatin immunoprecipitation-qPCR

Chromatin immunoprecipitation-qPCR (ChIP-qPCR) was used to evaluate ETS1 enrichment at the MYO1B promoter region. Experiments were performed using the CST SimpleChIP Enzymatic Chromatin IP Kit (Magnetic Beads, #9003) according to the manufacturer’s instructions. Cells were crosslinked and lysed, chromatin was enzymatically digested or sonicated, and ETS1 antibody was used for immunoprecipitation. Histone H3 served as a positive control and IgG as a negative control. Immunoprecipitated products were washed, eluted, reverse-crosslinked and purified before qPCR.

### 2.11 ETS1 virtual knockdown and predicted age change

To investigate the relationship between ETS1 perturbation and the single-cell age-clock signal, pericyte expression data were analyzed using scTenifoldKnk. ETS1 was specified as the knockout gene, which simulated ETS1 perturbation and generated a differential-regulation profile after manifold alignment (Osor et al., 2022). The differential-regulation profile was represented by gene-level z-scores. For each output record, the differential-regulation vector was correlated with the age-clock gene-effect vector over shared genes using Pearson correlation (ρ). To ensure consistent directional alignment, the differential-regulation profile was aligned using the age-clock genes with the largest absolute effects. The cell-type-level predicted change was then estimated by integrating three components: a global scaling factor (γ), the aligned correlation (ρ_aligned), and a cell-type-specific scaling parameter (S_CT). This value reflects the estimated shift in the age-clock signal specifically for the cell type under investigation. To translate this cell-type-level change into a predicted age change (ΔAge), two additional factors were incorporated: the contribution weight of that cell type in the age-clock meta-model (W_CT), and the observed prediction range for that cell type (D), which serves as a normalization factor. The resulting ΔAge thus represents a model-derived estimate of how ETS1 knockdown would shift the aging signal, accounting for both the strength of the regulatory perturbation and the relative importance of the affected cell type in the overall age-clock architecture. The complete calculation workflow is summarized in the accompanying formula diagram.

### 2.12 FDA drug-library screening, molecular docking and protein-ligand interaction profiling

An FDA drug library was used for virtual screening. Candidate small molecules underwent three-dimensional conformer generation and energy optimization before conversion to docking input formats, followed by molecular docking against ETS1. Docking was performed using AutoDock Vina, and candidate molecules were prioritized according to docking score, redocking consistency, ligand efficiency and interaction features (Trott and Olson, 2010; Forli et al., 2016). The Protein-Ligand Interaction Profiler (PLIP) was used to identify protein-ligand interaction types, including hydrogen bonds, hydrophobic interactions, pi-pi stacking and salt bridges (Salentin et al., 2015).

### 2.13 AlphaFold structure segmentation and molecular dynamics simulation

ETS1 structural analysis was performed using AlphaFold-predicted structures (Jumper et al., 2021; Varadi et al., 2022). The structure was segmented according to pLDDT, domain position, the DNA-binding domain, potential pocket-domain segments and flexible regions to avoid overinterpreting docking results from low-confidence or highly flexible regions. Candidate complexes were further analyzed by molecular dynamics simulation to evaluate complex stability, key-residue interactions, root-mean-square deviation (RMSD), root-mean-square fluctuation (RMSF), hydrogen-bond occupancy, residue distances and trajectory clustering. Simulations and trajectory analyses were performed using OpenMM, MDAnalysis (Eastman et al., 2017; Michaud‐Agrawal et al., 2011; Gowers et al., 2016).

### 2.14 OCTET/biolayer interferometry binding assay

OCTET/biolayer interferometry (BLI) was used to detect the binding response between ipragliflozin and recombinant ETS1 protein. Recombinant ETS1 protein was immobilized on the appropriate sensor, incubated with different concentrations of ipragliflozin, and real-time responses during association and dissociation were recorded.

### 2.15 D-gal-induced rat skin aging model and ipragliflozin intervention

Animal experiments were performed to evaluate the effect of ipragliflozin on D-Gal-induced skin aging model. Five-week-old male SD rats were used with 6 rats per group. D-galactose was administered subcutaneously at 200 mg/kg for 8 weeks (Umbayev et al., 2020). Ipragliflozin was prepared in 0.5% CMC and administered by oral gavage at 10 mg/kg/day (Salim et al., 2016). After the experiment, dorsal skin tissues were collected for H&E staining, epidermal-thickness assessment, IHC staining. All animal experiments were approved by the Animal Ethics Committee of Shengjing Hospital of China Medical University (2025PS331K) and conducted in accordance with GB/T 35892-2018 and the Guide for the Care and Use of Laboratory Animals; reporting follows the ARRIVE 2.0 guidelines.

### 2.16 Statistical analysis

Statistical analyses were performed using GraphPad Prism, R and Python. Continuous variables were presented as mean ± standard deviation or median with interquartile range according to data distribution. For *in vitro* experiments, three independent experiments were performed. Two-group comparisons were performed using t-test, and multiple-group comparisons were performed using one-way ANOVA. Pearson correlation was used for correlation analysis. Multiple comparisons were corrected using the Benjamini-Hochberg method where appropriate. P < 0.05 was considered statistically significant. Significance symbols in figures were defined as * represented p < 0.05, ** represented p < 0.01 and *** represented p < 0.001.

## 3 Results

### 3.1 The vascular niche carries major aging signals in a single-cell skin age-clock model

To identify the cell type that best reflect age-related change in the skin single-cell transcriptome, we first built an age-prediction framework based on expression profiles. Single-cell skin datasets were harmonized by cell-type annotation, aggregated into pseudo-bulk matrices per sample and cell type, and modeled by machine learning with cross-validation. Predicted age was calibrated against chronological age, and prediction performance was used to identify the cell types that contributed most to the skin aging signal (Figure 1A).

**FIGURE 1:**
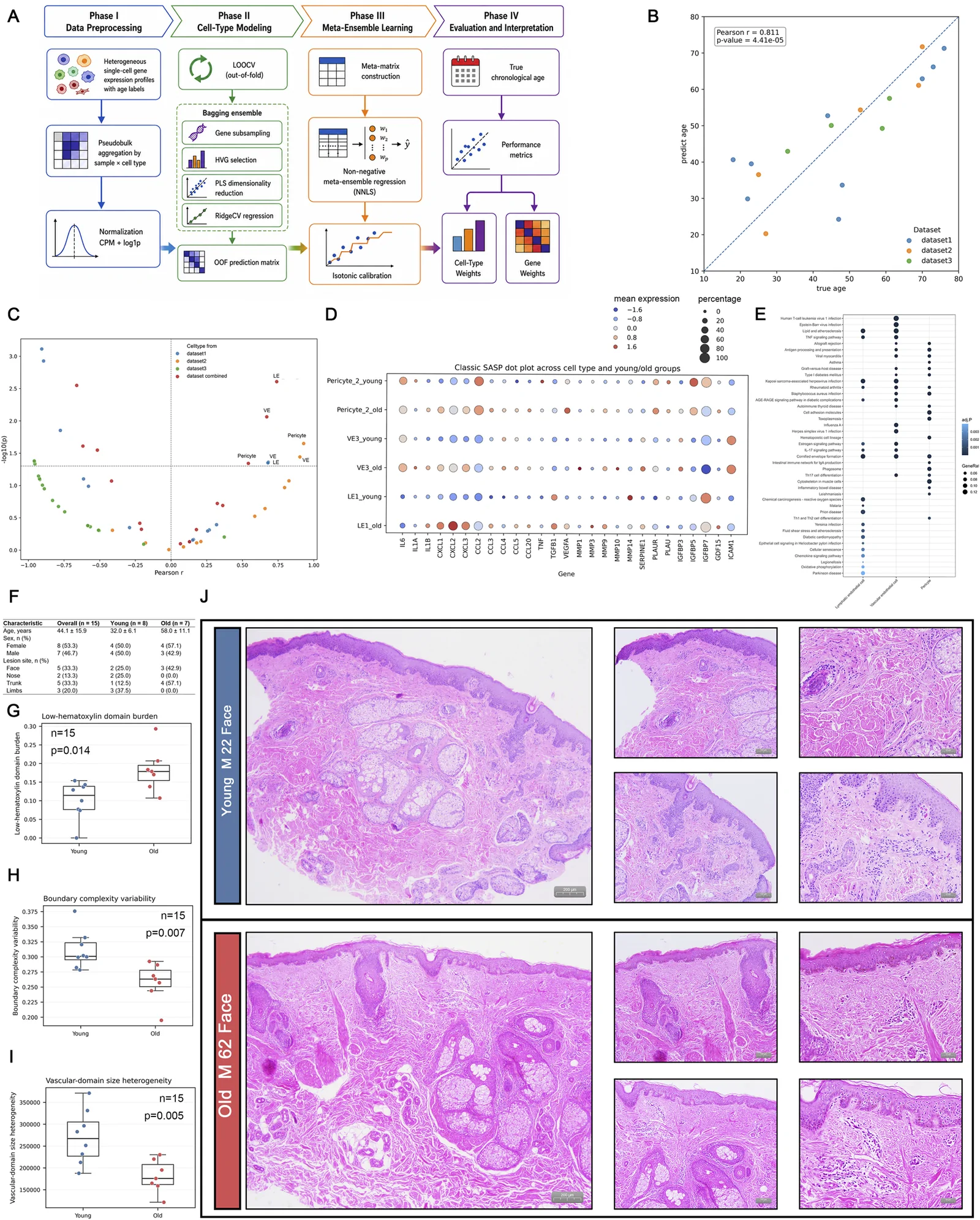
The vascular niche carries major aging signals in a single-cell skin age-clock model **(A)** Diagram of single-cell skin age-clock modeling. **(B)** Scatter plot of chronological age and predicted age across the three datasets. **(C)** Distribution of correlation between age prediction and chronological age for each cell type in individual and merged datasets. Dashed lines indicate r = 0 and p = 0.05; labeled LE, VE and pericytes are major positively correlated cell types. **(D)** Dot plot of SASP genes in LE, VE and Pericyte in young and old groups from the merged dataset. Dot size indicates the fraction of expressing cells and color indicates normalized average expression. **(E)** KEGG enrichment of age-clock-weighted genes. Dot size indicates GeneRatio and color indicates adjusted p value. **(F)** Clinical information table for local skin samples. **(G–I)** Digital H&E image quantification of **(G)** low-hematoxylin-area burden, **(H)** boundary-complexity variation and **(I)** vascular-area size heterogeneity. **(J)** Representative H&E images and local magnifications of young and old samples.

The model retained age-prediction capacity across the three datasets, with predicted age positively correlated with chronological age (Pearson r = 0.811, p = 4.41 × 10^−5^, n = 18; Figure 1B). When stratified by cell type, lymphatic endothelial cells (LE), vascular endothelial cells (VE), and pericytes recurred among the positively correlated cell types. In dataset 1, VE (r = 0.681, p = 0.0435, n = 9) and LE (r = 0.678, p = 0.0445, n = 9) correlated with age; in dataset 2, VE (r = 0.929, p = 0.0224, n = 5) and Pericyte (r = 0.902, p = 0.0361, n = 5) correlated with age; and in the merged data, LE, VE, and Pericyte remained the main positively correlated signals (Figure 1C). In the merged data, SASP gene dot plots showed differences in several inflammatory and senescence-related genes between young and old groups in VE-, LE-, and pericyte-associated subclusters (Figure 1D), and KEGG enrichment of age-clock-weighted genes covered cellular senescence, inflammation, viral infection, and antigen-presentation pathways (Figure 1E). The vascular niche identified by the age-clock model therefore contributed to age prediction and was also associated with SASP and immune-inflammatory pathways.

To test whether these single-cell findings corresponded to observable tissue changes, we examined 15 skin samples (8 young, 7 old; Figure 1F). Digital analysis of H&E images showed a higher low-hematoxylin-area burden in old samples (p = 0.014; Figure 1G), reduced variation in boundary complexity (p = 0.007; Figure 1H), and reduced heterogeneity in vascular-area size (p = 0.005; Figure 1I). Representative H&E images showed visible differences between young and old samples in the epidermis, dermal collagen, and perivascular structures (Figure 1J). These histological findings support the vascular aging niche identified at the single-cell level.

### 3.2 MYO1B correlates with an aging-associated pericyte senescence subcluster

Among the vascular cell types, pericytes are closely involved in vessel wall stability and local microenvironment regulation, so we next analyzed whether lymphatic endothelial cells, vascular endothelial cells, and pericytes contained age-related subclusters. NMF clustering of these 3 cell types in two datasets identified three major pericyte clusters in each dataset (Figure 2A). Notably, Cluster 3 was enriched with cells from older donors. In contrast, VE and LE showed no clear age-related cluster distribution (Figure 2B,C; Supplementary Figure 1E, F). And sample proportion also indicated that Cluster 3 increased in old samples (Figure 2D). Analysis of NMF clusters and age correlation in VE, LE, and pericytes likewise supported a positive association between pericyte clusters and age (Supplementary Figure 1C).

**FIGURE 2:**
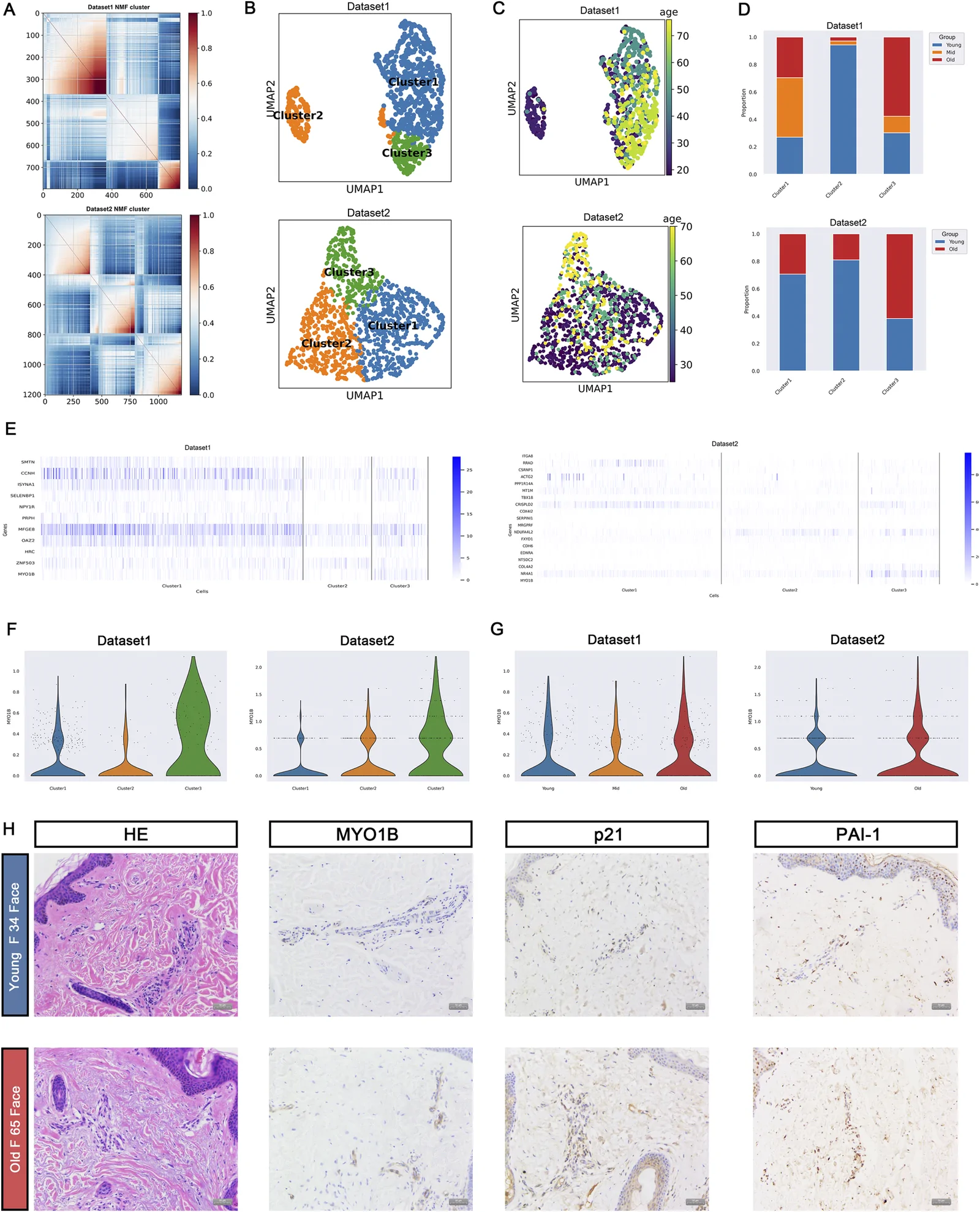
MYO1B correlates with an aging-associated pericyte senescence subcluster **(A)** NMF consensus cluster heatmaps of pericytes in two datasets. **(B)** UMAP showing spatial distributions of pericyte clusters 1-3 in dataset 1 and dataset 2. **(C)** UMAP showing age distribution in the datasets. **(D)** Sample-proportion plots of clusters across age groups. **(E)** Heatmap of cluster markers in two datasets. **(F)** Violin plots of MYO1B expression across pericyte clusters 1-3. **(G)** Violin plots of MYO1B expression across age groups. **(H)** Representative H&E, MYO1B IHC, p21 IHC and PAI-1 IHC images from young and old skin samples.

To find reliable molecular markers for these age-related clusters, we evaluated the consistency of cluster markers across both datasets. Differential expression analysis identified MYO1B as a common candidate marker in the aging-associated pericyte clusters of both datasets (Figure 2E; Supplementary Figure 1D). Consistently, MYO1B violin plots showed different expression across pericyte clusters and age groups, with more prominent expression in the older-age-associated cluster (Figures 2F,G). In local skin samples, old samples showed stronger MYO1B, p21, and PAI-1 immunohistochemical signals than young samples (Figure 2H).

### 3.3 Inflammatory stimulation induces a MYO1B-positive endothelial-to-pericyte-like transition

MYO1B immunohistochemistry showed expression specifically in vascular structures. To clarify the expression pattern of MYO1B across these vascular components, we performed a joint analysis of VE and pericyte Cluster 3 cells using the single-cell transcriptomic data. UMAP showed a continuous topological proximity between pericyte and VE, suggesting a possible continuous transition between the two states (Figure 3A).

**FIGURE 3:**
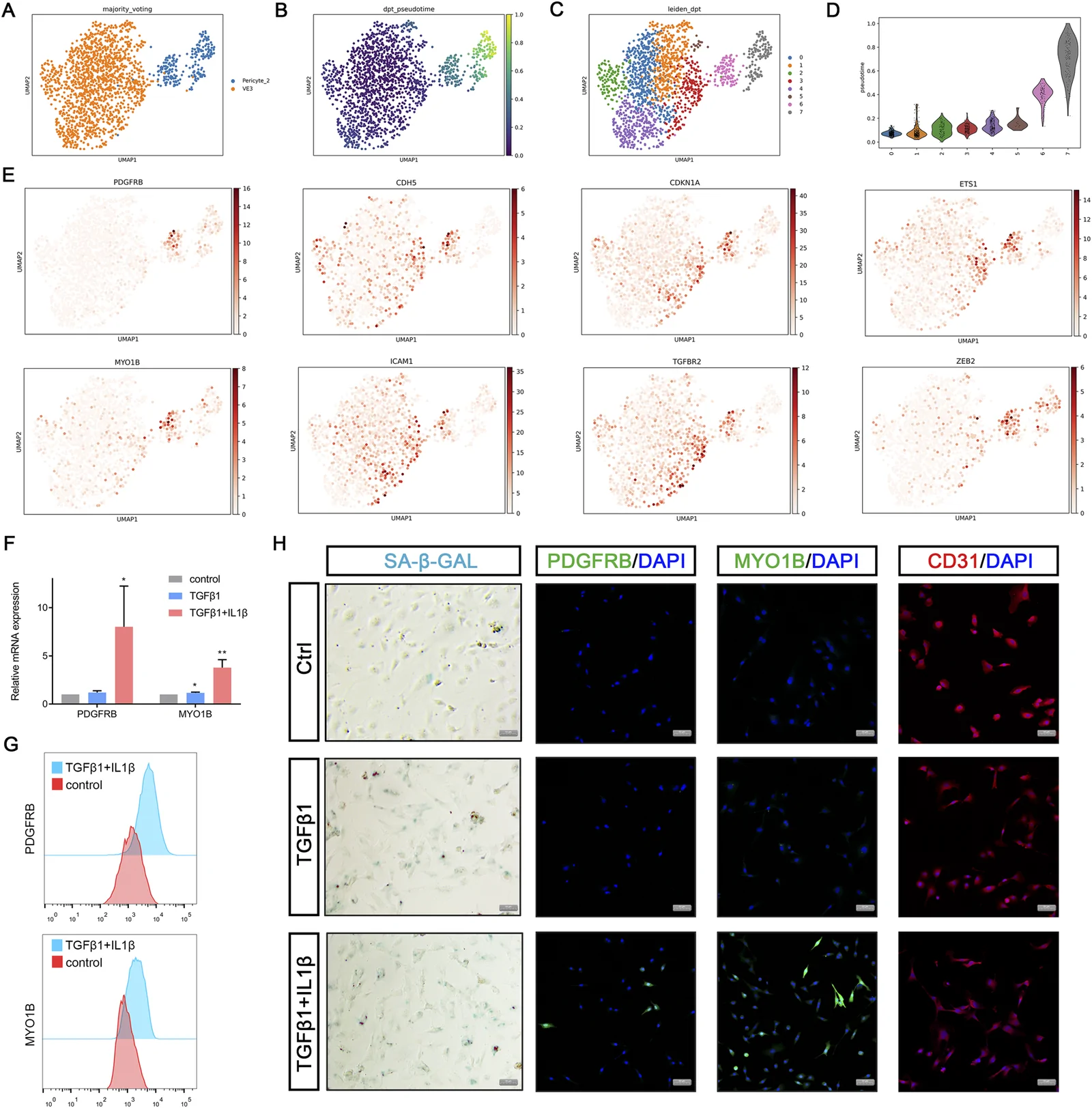
Inflammatory stimulation induces a MYO1B-positive endothelial-to-pericyte-like transition **(A)** Joint UMAP of VE3 and Pericyte_2-related cells in the merged dataset. **(B)** Distribution of diffusion pseudotime (DPT) in UMAP space. **(C)** Distribution of clusters reclustered according to DPT results. **(D)** Pseudotime violin plots for pseudotime-associated clusters. **(E)** Expression distributions of PDGFRB, CDH5, CDKN1A, ETS1, MYO1B, ICAM1, TGFBR2 and ZEB2 in the joint UMAP. **(F)** Relative mRNA expression of PDGFRB and MYO1B in HMEC-1 cells under control, TGF-β1 and TGF-β1+IL-1β. **(G)** Flow-cytometry distributions of PDGFRB and MYO1B in HMEC-1 cells comparing control and TGF-β1+IL-1β. **(H)** SA-β-gal staining and PDGFRB/DAPI, MYO1B/DAPI and CD31/DAPI immunofluorescence images under control, TGF-β1 and TGF-β1+IL-1β.

Pseudotime analysis revealed a continuous transcriptomic continuum along the UMAP (Figures 3B–D). Gene-expression mapping showed spatially heterogeneous distribution of pericyte, endothelial, senescence, inflammatory and EndoMT-related markers—PDGFRB, CDH5, CDKN1A, ETS1, MYO1B, ICAM1, TGFBR2, and ZEB2—with most of these markers appearing in parts of the transitional region (Figure 3E). These patterns suggest a continuous endothelial-to-pericyte-like transition linking endothelial cells, pericyte-like states, inflammatory responses, and senescence markers.

Based on the activation of inflammatory signals observed in our datasets and the role of TGF-β as a benchmark inducer of EndoMT, we next evaluated whether these conditions were sufficient to drive a similar phenotype *in vitro* using HMEC-1 cells. RT-qPCR showed that combined TGF-β1 and IL-1β treatment increased PDGFRB and MYO1B mRNA (Figure 3F). Flow cytometry also showed increased PDGFRB and MYO1B expression after TGF-β1/IL-1β treatment (Figure 3G). SA-β-gal staining and immunofluorescence revealed an increase in SA-β-gal-positive cells under TGF-β1/IL-1β conditions, whereas inflammatory stimulation specifically induced the upregulation of PDGFRB and MYO1B alongside reduced CD31 expression (Figure 3H). These results indicate that the endothelial-to-pericyte-like transition is closely associated with an inflammatory endothelial senescence phenotype.

### 3.4 ETS1 regulates MYO1B expression and induces cellular senescence

Having shown that inflammatory conditions can induce a MYO1B-positive endothelial-to-pericyte-like phenotype, we next screened for potential transcription factors regulating MYO1B expression. Correlation analysis between transcription-factor activity and MYO1B expression revealed that ETS1 was positively correlated with MYO1B expression, identifying ETS1 as a candidate upstream factor for MYO1B expression (Figure 4A).

**FIGURE 4:**
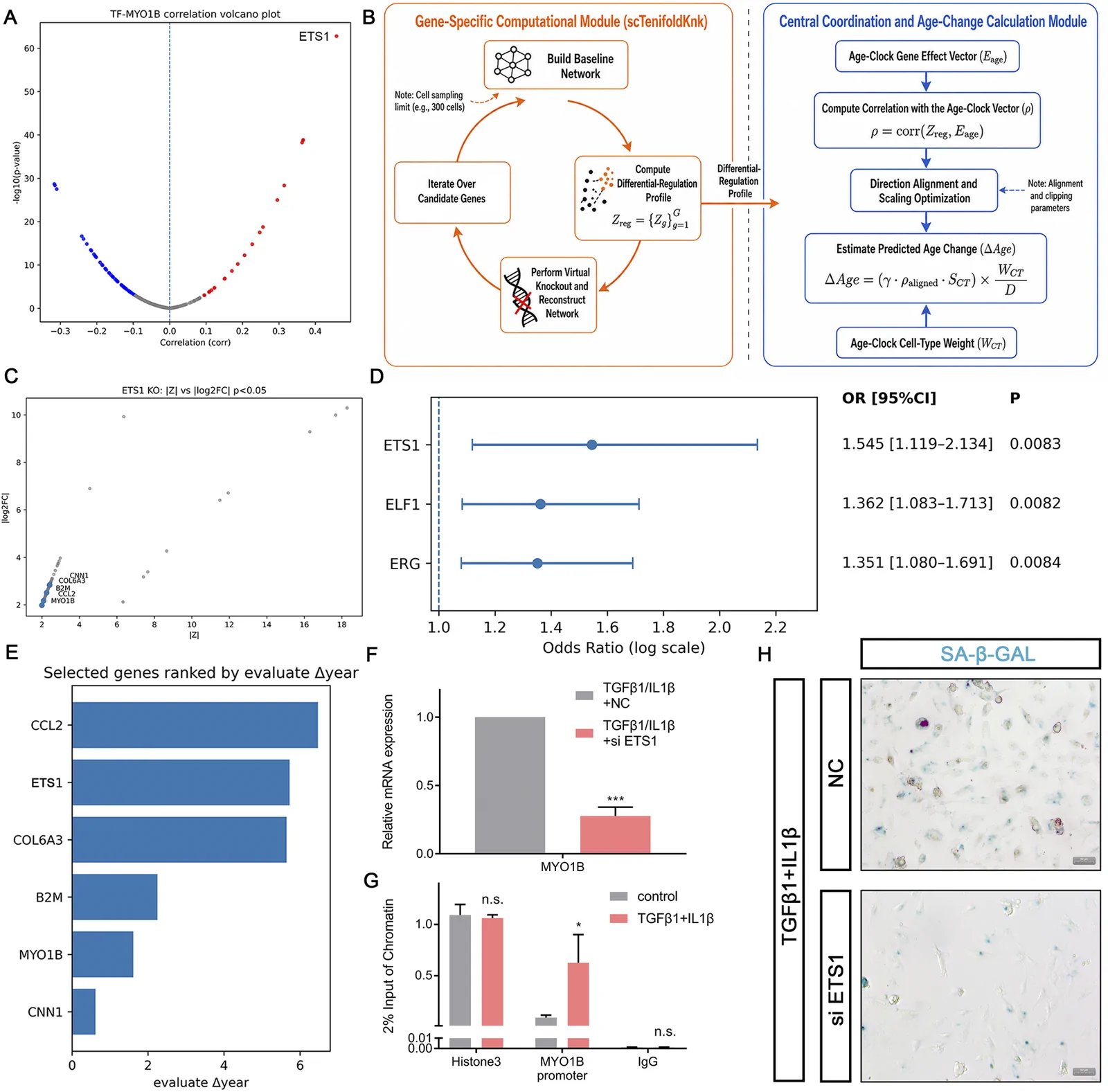
ETS1 regulates MYO1B expression and induces cellular senescence **(A)** TF regulon-MYO1B correlation volcano plot showing the correlation between transcription-factor activity and MYO1B expression. **(B)** Diagram of the scTenifoldKnk virtual-knockdown and predicted-age-change module. **(C)** Differential-regulation scatter plot after virtual ETS1 knockout. **(D)** Forest plot of age-associated odds ratios for ETS1, ELF1 and ERG in the age-clock model. **(E)** Bar plot ranking ETS1 and inferred downstream genes by predicted age change after virtual knockdown. **(F)** Effect of siETS1 on MYO1B mRNA under TGF-β1/IL-1β. **(G)** ChIP-qPCR showing ETS1 enrichment at the MYO1B promoter; histone H3 and IgG were used as controls. **(H)** Representative SA-β-gal images of NC and siETS1 groups under TGF-β1/IL-1β.

We further integrated scTenifoldKnk virtual-knockdown model with our age-prediction model to assess how regulating ETS1 impacts age-clock change. The workflow simulated candidate gene knockdown in the cell network to derive a differential regulation profile, and combined it with the age-clock weight vector to estimate predicted age change (Figure 4B). After virtual ETS1 knockout, aging- and EndoMT-related genes including MYO1B, CCL2, COL6A3, B2M, and CNN1 showed significant differential regulation (Figure 4C). Consistent with this, the age-clock model forest plot showed positive associations between age-related status and the ETS-family genes ETS1 (OR = 1.545, 95% CI: 1.119–2.134, p = 0.0083), ELF1 (OR = 1.362, 95% CI: 1.083–1.713, p = 0.0082), and ERG (OR = 1.351, 95% CI: 1.080–1.691, p = 0.0084) (Figure 4D). In the predicted-age-change ranking from virtual knockdown, CCL2, ETS1, COL6A3, B2M, and MYO1B substantially affected model-predicted age change (Figure 4E), further supporting a close relationship between ETS1 and age-related gene networks.

To further validate the relationship between ETS1 and MYO1B, we performed siETS1 intervention under TGF-β1/IL-1β conditions. ETS1 knockdown reduced MYO1B mRNA expression (Figure 4F). ChIP-qPCR analysis confirmed the binding of ETS1 to the MYO1B promoter and revealed a significant increase in ETS1 enrichment upon TGF-β1/IL-1β stimulation (Figure 4G). Also, siETS1 reduced SA-β-gal-positive staining (Figure 4H). Together, these results identify ETS1 as an upstream regulator of MYO1B, which drives the development of the endothelial-to-pericyte-like senescence phenotype.

### 3.5 Identification of ipragliflozin as a potential modulator of ETS1-MYO1B axis

Having established ETS1 as a key upstream regulator, we next screened for small molecules that could modulate the ETS1–MYO1B axis. ETS1 contains several transcription-regulatory regions harboring potential drug-binding pockets. We therefore used virtual screening of an FDA drug library to identify small molecules that might interact with these regions (Figure 5A). Molecular docking identified 17 candidate molecules with high binding potential, with lumacaftor, vibegron, and ipragliflozin ranking in the top three (Figure 5B). PLIP interaction profiles showed hydrophobic contacts, hydrogen bonds, π-stacking, π-cation interactions, salt bridges, and halogen bonds between the candidates and ETS1 (Figure 5C).

**FIGURE 5:**
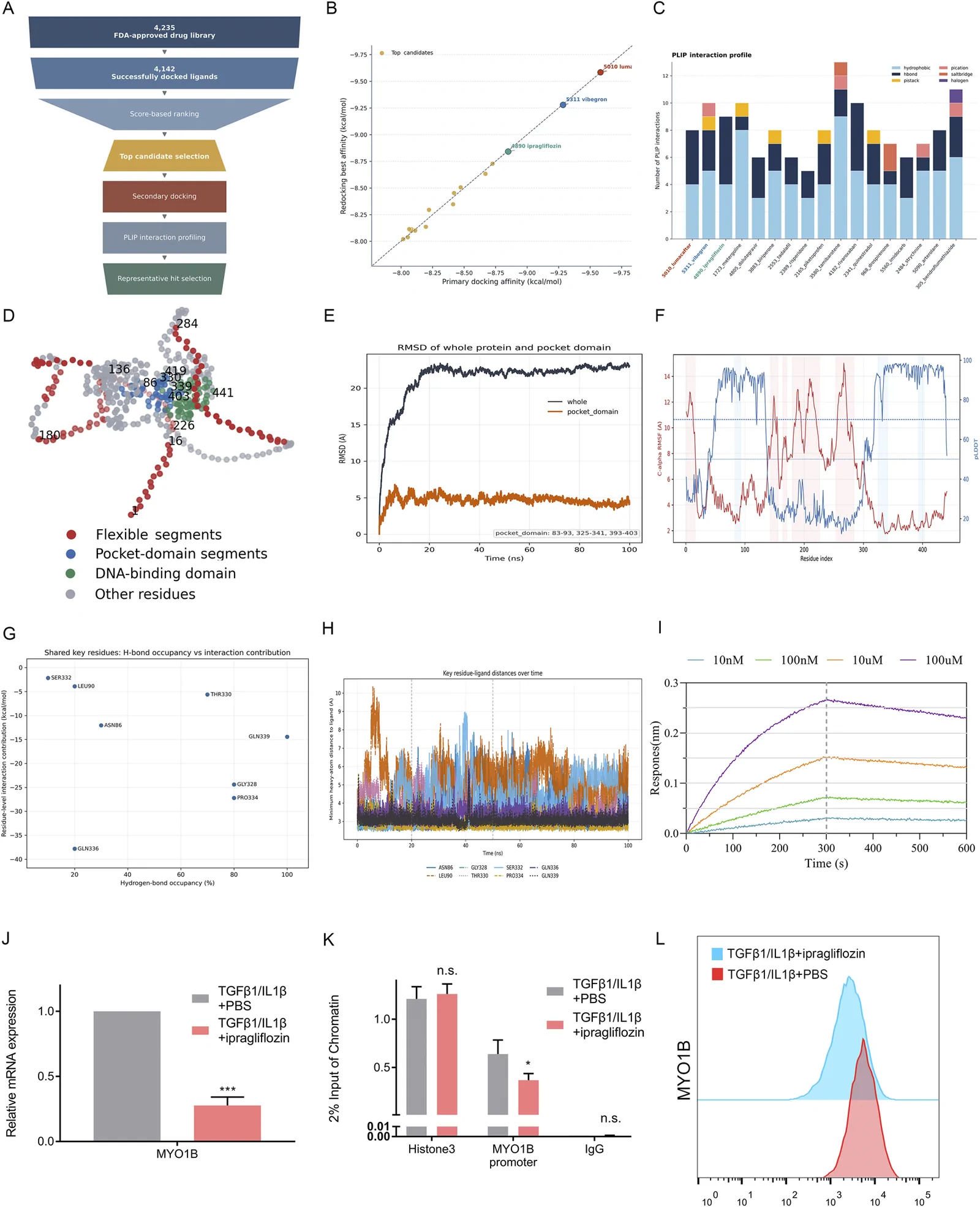
Identification of Ipragliflozin as a potential modulator of ETS1-MYO1B axis **(A)** ETS1 virtual-screening workflow. **(B)** Candidate ranking by primary docking affinity and redocking best affinity, with the top candidates labeled. **(C)** PLIP interaction profile, stacked bars show hydrophobic, hydrogen-bond, pi-stacking, pi-cation, salt-bridge and halogen interactions for candidate molecules. **(D)** Flexible segments, pocket-domain segments, DNA-binding domain and other residues in the AlphaFold-predicted ETS1 structure. **(E)** RMSD of the whole protein and pocket domain during MD. The pocket domain was defined as residues 83-93, 325-341 and 393-403. **(F)** Integrated residue C-alpha RMSF and pLDDT plot for simultaneous assessment of residue flexibility and structure-prediction confidence. **(G)** Integrated plot of hydrogen-bond occupancy and MM/GBSA residue contribution for the shared key residues SER332, LEU90, ASN86, THR330, GLN339, GLY328, PRO334 and GLN336. **(H)** Minimum heavy-atom distance fluctuations from the shared key residues to the ligand over time. **(I)** OCTET detection of binding responses between ipragliflozin and ETS1 at different concentrations. **(J)** Effect of ipragliflozin on MYO1B mRNA under TGF-β1/IL-1β. **(K)** Effect of ipragliflozin on MYO1B promoter ChIP-qPCR. **(L)** Effect of ipragliflozin on MYO1B flow-cytometry signal.

To further evaluate these interactions, we annotated the flexible loops and functional domains based on the AlphaFold-predicted full-length ETS1 (Figure 5D). Since low-confidence (low-pLDDT) regions often represent intrinsically disordered segments that distort full-length docking, ligands targeting high-confidence pockets yield far greater interpretive value. Further MD analysis was used to assess the dynamic stability of the ETS1–ipragliflozin complex. RMSD curves showed large fluctuations and poorer stability for the whole-structure simulation, whereas the local trajectories of the pocket domains showed robust conformational stability (Figure 5E). The local pocket-domain trajectories showed stable conformation during simulation, supporting a potential interaction between ipragliflozin and ETS1 transcription-regulatory region. Integrated residue RMSF and pLDDT analysis further indicated that ipragliflozin potentially binds the ETS1 region at residues 320–340 (Figure 5F).

At the residue level, integration of hydrogen-bond occupancy and MM/GBSA energy decomposition identified GLN336, PRO334, GLN339, GLY328, THR330, ASN86, LEU90, ALA327, TYR329, and SER332 as the key energetic hotspots contributing to the stability of the ETS1–ipragliflozin complex (Figure 5G). Tracking the minimum heavy-atom distances over time demonstrated that ipragliflozin maintained persistent, close-range contacts with GLN339, GLN336, THR330, ASN86, and SER332 throughout the simulation trajectory, reinforcing their role in coordinating ligand binding (Figure 5H).

To validate the predictions, biolayer interferometry (BLI) via OCTET system was performed, revealing a clear concentration-dependent binding between ipragliflozin and ETS1 (Figure 5I). Functionally, under TGF-β1/IL-1β conditions, ipragliflozin treatment significantly downregulated the mRNA expression of MYO1B (Figure 5J). Concurrently, ChIP-qPCR revealed diminished MYO1B promoter binding (Figure 5K), while FACS analysis confirmed reduced MYO1B expression (Figure 5L). These results support ipragliflozin as a candidate modulator of the ETS1–MYO1B axis.

### 3.6 Ipragliflozin reduces skin aging *in vitro* and *in vivo*

To determine whether this candidate molecule affected the endothelial-to-pericyte-like transition and senescence phenotype, we first evaluated its protective effects in the inflammatory HMEC-1 cell model. Under TGF-β1/IL-1β conditions, ipragliflozin treatment decreased the expression of PDGFRB (Figure 6A) and reduced SA-β-gal-positive senescent cells (Figure 6B). RT-qPCR analysis further confirmed that ipragliflozin effectively suppressed the expression of key senescence-associated genes, specifically CDKN1A/p21 and SERPINE1/PAI-1 (Figure 6C). Consistent with the downregulation of MYO1B described above, these changes confirm that ipragliflozin can successfully abrogate inflammation-driven endothelial senescence and the subsequent pericyte-like transition.

**FIGURE 6:**
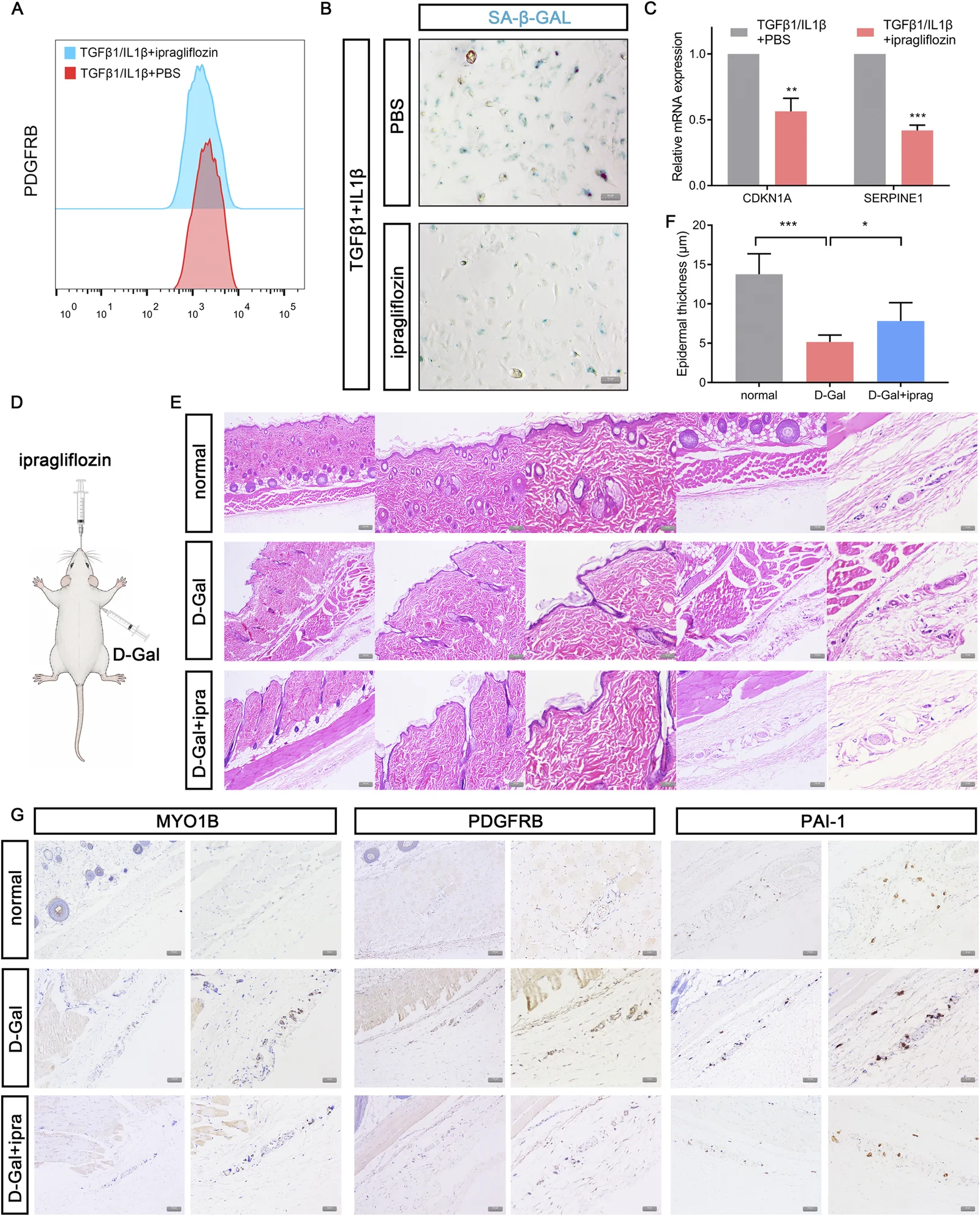
Ipragliflozin reduces skin aging *in vitro* and *in vivo* **(A)** PDGFRB flow-cytometry distributions under ipragliflozin treatment. **(B)** SA-β-gal staining under ipragliflozin treatment. **(C)** Effects of ipragliflozin on relative CDKN1A and SERPINE1 mRNA expression. **(D)** Diagram of the rat dorsal-skin D-Gal-induced aging model and ipragliflozin oral-gavage intervention. **(E)** Representative dorsal-skin H&E images from normal, D-Gal and D-Gal + ipragliflozin groups. **(F)** Epidermal-thickness quantification across the three groups. **(G)** Representative MYO1B, PDGFRB, PAI-1 IHC images from the three groups.

To assess these findings *in vivo*, we established a rat model of D-galactose (D-Gal)-induced skin aging and subsequently administered ipragliflozin via oral gavage (Figure 6D). Representative H&E staining showed altered skin architecture and a prominent loss of epidermal thickness in the D-Gal-induced group; remarkably, ipragliflozin intervention substantially rescued these structural degenerations and restored epidermal thickness (Figures 6E,F). Further IHC results showed that the therapeutic effects of ipragliflozin *in vivo* were characterized by a prominent reduction in MYO1B and PDGFRB expression, accompanied by a decrease in PAI-1 within the perivascular regions (Figure 6G). Collectively, these *in vitro* and *in vivo* results identified ipragliflozin as a powerful therapeutic agent against skin aging by downregulating the ETS1-MYO1B regulatory axis.

## 4 Discussion

In this study, we find that the cutaneous vascular niche serves as a critical component for aging-associated signatures, which are tightly linked to EndoPT. Our single-cell skin age-clock model identified that senescent and inflammatory programs are enriched across vascular endothelial and perivascular populations. Within this niche, we found an aging-associated pericyte subcluster characterized by MYO1B expression. Trajectory analysis delineated a progressive transition linking endothelial states to a pericyte-like phenotype under inflammatory conditions. We show that ETS1 transcriptionally regulates MYO1B to drive this inflammatory senescent phenotype. Furthermore, target-engaged screening identified the SGLT2 inhibitor ipragliflozin as a potent rescue agent, which effectively suppresses the ETS1-MYO1B axis and significantly ameliorates vascular aging in skin.

By utilizing machine learning to construct a predictive biological indicator, our age clock uncovers the cutaneous microvascular niche as a primary focus of transcriptomic aging. Rather than aging across dermis, the senescent and inflammatory programs captured by our single-cell clock within endothelial and perivascular populations, suggesting that the vascular niche functions as a local amplifier of age-related molecular changes. Physiologically, vascular cells serve as primary channels for molecular transport and exchange; they are uniquely positioned to intercept and amplify senescent factors generated by chronic photoaging, localized immune stress, and systemic metabolic remodeling. These findings support recent skin aging studies showing that vascular cells experience major transcriptomic changes over time (Zou et al., 2021; Solé-Boldo et al., 2020).

A central finding of this study is that aging skin cells undergo a gradual shift. Specifically, our data trace a progressive continuum connecting endothelial states to an aging-associated pericyte-like subcluster marked by MYO1B expression. We refer to this process as endothelial-to-pericyte transition (EndoPT) (Chen et al., 2016). Computational trajectory analysis indicates that under chronic inflammation, endothelial cells lose their original identity and acquire perivascular traits, including classic pericyte markers and mesenchymal components. At the same time, these transforming cells upregulate cell-cycle arrest genes and SASP factors. This synchronous change suggests that mesenchymal remodeling and cellular senescence are connected within the inflamed vascular niche. We view EndoPT as a flexible phenotypic drift, though whether this transition can be reversed *in vivo* remains a question to be addressed by future spatial transcriptomics and genetic lineage tracing.

Previous studies suggest that ETS1 participates in vascular inflammation and cellular stress responses (Zhan et al., 2005). In this study, we identified ETS1 as the key transcription factor that connects chronic inflammation to the EndoPT transition. We show that inflammatory stress prompts ETS1 to bind directly to the MYO1B promoter, an interaction that remains weak under healthy conditions. This makes ETS1 a connection between inflammatory stimulation, endothelial changes and vessel-wall remodeling. Crucially, recent findings that inflammation and senescence remodel the epigenomic landscape, particularly by shifting chromatin accessibility at ETS-family binding motifs, further support our observations (Teruyama et al., 2001). MYO1B has been proposed as a specific pericyte marker with better discrimination from vascular smooth muscle cells and fibroblasts than conventional markers (Meguro et al., 2017). Other studies have used MYO1B alongside PDGFRB, RGS5 and ABCC9 to define pericyte identities, revealing significant transcriptional heterogeneity across different tissues (Potts et al., 2025; Baek et al., 2022). Here, we demonstrate that MYO1B induction coordinates both cytoskeletal changes linked to endothelial senescence (Yu et al., 2023; McConnell and Tyska, 2010) and the activation of a pericyte-like program. These findings indicate that the ETS1-MYO1B axis functions as an inflammation-driven mediator to connect EndoPT with cellular senescence.

The identification of ipragliflozin to target the ETS1-MYO1B axis highlights the therapeutic potential of targeting vascular niche aging. SGLT2 inhibitors were originally designed to lower blood glucose in type 2 diabetes. However, clinical trials have shown they offer cardiovascular and renal protection that cannot be explained by blood sugar control alone. This has sparked interest in their glucose-independent, pleiotropic effects, such as reducing oxidative stress and dampening tissue inflammation (Dimitriadis et al., 2023). Regarding their anti-aging properties, canagliflozin has been reported to reduce pathological senescent-cell burden (Katsuumi et al., 2024), dapagliflozin can attenuate diabetes-related endothelial senescence through the SIRT1 pathway (Tai et al., 2023), and ipragliflozin can ameliorate endothelial dysfunction in diabetic mice (Salim et al., 2016). Our study extends these findings by showing that ipragliflozin reduced ETS1-mediated MYO1B expression, thereby reversing the senescent pericyte-like phenotype in both endothelial cell lines and aged rat skin. These results indicate that ipragliflozin mediates localized vascular protective effect by targeting vascular niche aging under normoglycemic conditions. Consequently, our findings support the therapeutic repurposing of this FDA drug within current longevity research to expedite clinical translation.

Several limitations of this study warrant consideration. First, the limited single-cell datasets focused on skin aging restricted our ability to perform extensive external validation. Future expansion of tissue-specific aging atlases will help confirm our findings. Second, resolving EndoPT at the tissue level remains challenging through standard histological staining alone, as transitional cells share overlapping markers and close spatial proximity with both native endothelial cells and pericytes. Further work integrating spatial transcriptomics and genetic fate mapping will be essential to directly visualize these dynamics *in vivo*. Finally, while we identified ipragliflozin as a promising agent to mitigate this vascular degeneration, our mechanistic exploration remains preliminary. The exact molecular binding sites of ipragliflozin and pharmacokinetics in aging tissue require further validation.

In summary, our study establishes a direct link between chronic microvascular inflammation, cellular senescence, and vascular wall cell plasticity during skin aging. By characterizing the EndoPT transition, our findings reveal that the vascular niche undergoes a progressive state shift during senescent degeneration. Overall, this study provides an integrated understanding of microvascular wall dynamics during skin aging, and offers a translational strategy for future research into vascular niche aging.

## Data Availability

The original contributions presented in the study are included in the article/Supplementary Material, further inquiries can be directed to the corresponding authors.

## Supplementary material

SUPPLEMENTARY FIGURE 1
**(A)** Beeswarm plot showing correlations between weighted genes and age among cell types, including VE, LE and pericytes. **(B)** Overall cell-interaction networks for pericytes, VE, LE and fibroblasts in two positively correlated datasets; edges and values indicate visualized interaction strength. **(C)** Pearson correlation heatmap between cNMF clusters and age in VE, LE and pericytes. **(D)** Venn diagram of pericyte cluster-specific genes in two datasets; MYO1B is located in the intersection. **(E)** cNMF clustering, age distribution and cluster distribution of VE cells in two datasets. **(F)** cNMF clustering, age distribution and cluster distribution of LE cells in two datasets.

## References

[B1] Armulik A. Genové G. Betsholtz C. (2011). Pericytes: developmental, physiological, and pathological perspectives, problems, and promises. Dev. Cell 21, 193–215. 10.1016/j.devcel.2011.07.001 21839917

[B2] Baek S. H. Maiorino E. Kim H. Glass K. Raby B. A. Yuan K. (2022). Single cell transcriptomic analysis reveals organ specific pericyte markers and identities. Front. Cardiovasc Med. 9, 876591. 10.3389/fcvm.2022.876591 35722109 PMC9199463

[B3] Bankhead P. Loughrey M. B. Fernández J. A. Dombrowski Y. McArt D. G. Dunne P. D. (2017). QuPath: open source software for digital pathology image analysis. Sci. Rep. 7, 16878. 10.1038/s41598-017-17204-5 29203879 PMC5715110

[B4] Basisty N. Kale A. Jeon O. H. Kuehnemann C. Payne T. Rao C. (2020). A proteomic atlas of senescence-associated secretomes for aging biomarker development. PLoS Biol. 18, e3000599. 10.1371/journal.pbio.3000599 31945054 PMC6964821

[B5] Bentov I. Reed M. J. (2015). The effect of aging on the cutaneous microvasculature. Microvasc. Res. 100, 25–31. 10.1016/j.mvr.2015.04.004 25917013 PMC4461519

[B6] Browaeys R. Saelens W. Saeys Y. (2020). NicheNet: modeling intercellular communication by linking ligands to target genes. Nat. Methods 17, 159–162. 10.1038/s41592-019-0667-5 31819264

[B7] Buckley M. T. Sun E. D. George B. M. Liu L. Schaum N. Xu L. (2023). Cell-type-specific aging clocks to quantify aging and rejuvenation in neurogenic regions of the brain. Nat. Aging 3, 121–137. 10.1038/s43587-022-00335-4 37118510 PMC10154228

[B8] Chen Q. Zhang H. Liu Y. Adams S. Eilken H. Stehling M. (2016). Endothelial cells are progenitors of cardiac pericytes and vascular smooth muscle cells. Nat. Commun. 7, 12422. 10.1038/ncomms12422 27516371 PMC4990645

[B9] Chung J. H. Eun H. C. (2007). Angiogenesis in skin aging and photoaging. J. Dermatol 34, 593–600. 10.1111/j.1346-8138.2007.00341.x 17727362

[B10] Coppé J. P. Patil C. K. Rodier F. Sun Y. Muñoz D. P. Goldstein J. (2008). Senescence-associated secretory phenotypes reveal cell-nonautonomous functions of oncogenic RAS and the p53 tumor suppressor. PLoS Biol. 6, e301–e2868. 10.1371/journal.pbio.0060301 19053174 PMC2592359

[B11] Dimitriadis K. Adamopoulou E. Pyrpyris N. Sakalidis A. Leontsinis I. Manta E. (2023). The effect of SGLT2 inhibitors on the endothelium and the microcirculation: from bench to bedside and beyond. Eur. Heart J. Cardiovasc Pharmacother. 9, 741–757. 10.1093/ehjcvp/pvad053 37500266

[B12] Domínguez C. C. Xu C. Jarvis L. B. Rainbow D. B. Wells S. B. Gomes T. (2022). Cross-tissue immune cell analysis reveals tissue-specific features in humans. Science 376, eabl5197. 10.1126/science.abl5197 35549406 PMC7612735

[B13] Eastman P. Swails J. Chodera J. D. McGibbon R. T. Zhao Y. Beauchamp K. A. (2017). OpenMM 7: rapid development of high performance algorithms for molecular dynamics. PLoS Comput. Biol. 13, e1005659. 10.1371/journal.pcbi.1005659 28746339 PMC5549999

[B14] Farage M. A. Miller K. W. Elsner P. Maibach H. I. (2008). Intrinsic and extrinsic factors in skin ageing: a review. Int. J. Cosmet. Sci. 30, 87–95. 10.1111/j.1468-2494.2007.00415.x 18377617

[B15] Forli S. Huey R. Pique M. E. Sanner M. F. Goodsell D. S. Olson A. J. (2016). Computational protein-ligand docking and virtual drug screening with the AutoDock suite. Nat. Protoc. 11, 905–919. 10.1038/nprot.2016.051 27077332 PMC4868550

[B16] Gayoso A. Lopez R. Xing G. Boyeau P. Valiollah Pour Amiri V. Hong J. (2022). A python library for probabilistic analysis of single-cell omics data. Nat. Biotechnol. 40, 163–166. 10.1038/s41587-021-01206-w 35132262

[B17] Gowers R. Linke M. Barnoud J. Reddy T. Melo M. Seyler S. (2016). “MDAnalysis: a python package for the rapid analysis of molecular dynamics simulations,” in Proceedings of the Python in Science Conference, 98–105.

[B18] Gu Z. Eils R. Schlesner M. (2016). Complex heatmaps reveal patterns and correlations in multidimensional genomic data. Bioinformatics 32, 2847–2849. 10.1093/bioinformatics/btw313 27207943

[B19] Haghverdi L. Büttner M. Wolf F. A. Buettner F. Theis F. J. (2016). Diffusion pseudotime robustly reconstructs lineage branching. Nat. Methods 13, 845–848. 10.1038/nmeth.3971 27571553

[B20] Hao Y. Hao S. Andersen-Nissen E. Mauck W. M. Zheng S. Butler A. (2021). Integrated analysis of multimodal single-cell data. Cell 184, 3573–3587.e29. 10.1016/j.cell.2021.04.048 34062119 PMC8238499

[B21] Harris C. R. Millman K. J. van der Walt S. J. Gommers R. Virtanen P. Cournapeau D. (2020). Array programming with NumPy. Nature 585, 357–362. 10.1038/s41586-020-2649-2 32939066 PMC7759461

[B22] Horvath S. (2013). DNA methylation age of human tissues and cell types. Genome Biol. 14, R115. 10.1186/gb-2013-14-10-r115 24138928 PMC4015143

[B23] Hunter J. D. (2007). Matplotlib: a 2D graphics environment. Comput. Sci. Eng. 9, 90–95. 10.1109/mcse.2007.55

[B24] Jassal B. Matthews L. Viteri G. Gong C. Lorente P. Fabregat A. (2020). The reactome pathway knowledgebase. Nucleic Acids Res. 48, D498–D503. 10.1093/nar/gkz1031 31691815 PMC7145712

[B25] Jumper J. Evans R. Pritzel A. Green T. Figurnov M. Ronneberger O. (2021). Highly accurate protein structure prediction with AlphaFold. Nature 596, 583–589. 10.1038/s41586-021-03819-2 34265844 PMC8371605

[B26] Kanehisa M. Goto S. (2000). KEGG: kyoto encyclopedia of genes and genomes. Nucleic Acids Res. 28, 27–30. 10.1093/nar/28.1.27 10592173 PMC102409

[B27] Katsuumi G. Shimizu I. Suda M. Yoshida Y. Furihata T. Joki Y. (2024). SGLT2 inhibition eliminates senescent cells and alleviates pathological aging. Nat. Aging 4, 926–938. 10.1038/s43587-024-00642-y 38816549 PMC11257941

[B28] Kotliar D. Veres A. Nagy M. A. Tabrizi S. Hodis E. Melton D. A. (2019). Identifying gene expression programs of cell-type identity and cellular activity with single-cell RNA-Seq. eLife 8, e43803. 10.7554/eLife.43803 31282856 PMC6639075

[B29] Lee D. D. Seung H. S. (1999). Learning the parts of objects by non-negative matrix factorization. Nature 401, 788–791. 10.1038/44565 10548103

[B30] Liberzon A. Birger C. Thorvaldsdóttir H. Ghandi M. Mesirov J. P. Tamayo P. (2015). The molecular signatures database hallmark gene set collection. Cell Syst. 1, 417–425. 10.1016/j.cels.2015.12.004 26771021 PMC4707969

[B31] López-Otín C. Blasco M. A. Partridge L. Serrano M. Kroemer G. (2023). Hallmarks of aging: an expanding universe. Cell 186, 243–278. 10.1016/j.cell.2022.11.001 36599349

[B32] Low E. Alimohammadiha G. Smith L. A. Costello L. F. Przyborski S. A. von Zglinicki T. (2021). How good is the evidence that cellular senescence causes skin ageing? Ageing Res. Rev. 71, 101456. 10.1016/j.arr.2021.101456 34487917 PMC8524668

[B33] Ma J. Sanchez-Duffhues G. Goumans M. J. ten Dijke P. (2020). TGF-β-Induced endothelial to mesenchymal transition in disease and tissue engineering. Front. Cell Dev. Biol. 8, 260. 10.3389/fcell.2020.00260 32373613 PMC7187792

[B34] McConnell R. E. Tyska M. J. (2010). Leveraging the membrane-cytoskeleton interface with myosin-1. Trends Cell Biol. 20, 418–426. 10.1016/j.tcb.2010.04.004 20471271 PMC2897960

[B35] McInnes L. Healy J. Melville J. (2018). UMAP: uniform manifold approximation and projection for dimension reduction. arXiv.

[B36] McKinney W. (2010). “Data structures for statistical computing in python,” in Proceedings of the Python in Science Conference, 56–61.

[B37] Meguro S. Akamatsu T. Matsushima S. Kosugi I. Kawasaki H. Arai Y. (2017). Phenotypic characterization of perivascular myoid cell neoplasms, using myosin 1B, a newly identified human pericyte marker. Hum. Pathol. 62, 187–198. 10.1016/j.humpath.2016.12.024 28088345

[B38] Michaud‐Agrawal N. Denning E. J. Woolf T. B. Beckstein O. (2011). MDAnalysis: a toolkit for the analysis of molecular dynamics simulations. J. Comput. Chem. 32, 2319–2327. 10.1002/jcc.21787 21500218 PMC3144279

[B39] Osorio D. Zhong Y. Li G. Huang J. Z. Cai J. J. Tian Y. (2022). scTenifoldKnk: an efficient virtual knockout tool for gene function predictions via single-cell gene regulatory network perturbation. Patterns (N Y) 3, 100434. 10.1016/j.patter.2022.100434 35510185 PMC9058914

[B40] Pedregosa F. Varoquaux G. Gramfort A. Michel V. Thirion B. Grisel O. (2011). Scikit-learn: machine learning in python. J. Mach. Learn Res. 12, 2825–2830.

[B41] Piera-Velazquez S. Jimenez S. A. (2012). Molecular mechanisms of endothelial to mesenchymal cell transition (EndoMT) in experimentally induced fibrotic diseases. Fibrogenes. Tissue Repair 5, S7. 10.1186/1755-1536-5-S1-S7 23259736 PMC3368755

[B42] Potts C. M. Yang X. Lynes M. D. Malka K. Liaw L. (2025). Exploration of conserved human adipose subpopulations using targeted single-nuclei RNA sequencing data sets. J. Am. Heart Assoc. 14, e038465. 10.1161/jaha.124.038465 40094187 PMC12132619

[B43] Reynolds G. Vegh P. Fletcher J. Poyner E. F. M. Stephenson E. Goh I. (2021). Developmental cell programs are co-opted in inflammatory skin disease. Science 371, eaba6500. 10.1126/science.aba6500 33479125 PMC7611557

[B44] Rittie L. Fisher G. J. (2015). Natural and sun-induced aging of human skin. Cold Spring Harb. Perspect. Med. 5, a015370. 10.1101/cshperspect.a015370 25561721 PMC4292080

[B45] Salentin S. Schreiber S. Haupt V. J. Adasme M. F. Schroeder M. (2015). PLIP: fully automated protein-ligand interaction profiler. Nucleic Acids Res. 43, W443–W447. 10.1093/nar/gkv315 25873628 PMC4489249

[B46] Salim H. M. Fukuda D. Yagi S. Soeki T. Shimabukuro M. Sata M. (2016). Glycemic control with ipragliflozin, a novel selective SGLT2 inhibitor, ameliorated endothelial dysfunction in Streptozotocin-Induced diabetic mouse. Front. Cardiovasc Med. 3, 43. 10.3389/fcvm.2016.00043 27833913 PMC5080286

[B47] Saul D. Kosinsky R. L. Atkinson E. J. Doolittle M. L. Zhang X. LeBrasseur N. K. (2022). A new gene set identifies senescent cells and predicts senescence-associated pathways across tissues. Nat. Commun. 13, 4827. 10.1038/s41467-022-32552-1 35974106 PMC9381717

[B48] Seabold S. Perktold J. (2010). “Statsmodels: econometric and statistical modeling with python,” in Proceedings of the Python in Science Conference, 92–96.

[B49] Solé-Boldo L. Raddatz G. Schütz S. Mallm J. P. Rippe K. Lonsdorf A. S. (2020). Single-cell transcriptomes of the human skin reveal age-related loss of fibroblast priming. Commun. Biol. 3, 188. 10.1038/s42003-020-0922-4 32327715 PMC7181753

[B50] Tacutu R. Thornton D. Johnson E. Budovsky A. Barardo D. Craig T. (2018). Human ageing genomic resources: new and updated databases. Nucleic Acids Res. 46, D1083–D1090. 10.1093/nar/gkx1042 29121237 PMC5753192

[B51] Tai S. Zhou Y. Fu L. Ding H. Zhou Y. Yin Z. (2023). Dapagliflozin impedes endothelial cell senescence by activating the SIRT1 signaling pathway in type 2 diabetes. Heliyon 9, e19152. 10.1016/j.heliyon.2023.e19152 37664712 PMC10469571

[B52] Teruyama K. Abe M. Nakano T. Iwasaka-Yagi C. Takahashi S. Yamada S. (2001). Role of transcription factor Ets-1 in the apoptosis of human vascular endothelial cells. J. Cell Physiol. 188, 243–252. 10.1002/jcp.1112 11424091

[B53] Trott O. Olson A. J. (2010). AutoDock vina: improving the speed and accuracy of docking with a new scoring function, efficient optimization, and multithreading. J. Comput. Chem. 31, 455–461. 10.1002/jcc.21334 19499576 PMC3041641

[B54] Umbayev B. Askarova S. Almabayeva A. Saliev T. Masoud A. R. Bulanin D. (2020). Galactose-Induced skin aging: the role of oxidative stress. Oxid. Med. Cell Longev. 2020, 7145656. 10.1155/2020/7145656 32655772 PMC7317321

[B55] Varadi M. Anyango S. Deshpande M. Nair S. Natassia C. Yordanova G. (2022). AlphaFold protein structure database: massively expanding the structural coverage of protein-sequence space with high-accuracy models. Nucleic Acids Res. 50, D439–D444. 10.1093/nar/gkab1061 34791371 PMC8728224

[B56] Virshup I. Rybakov S. Theis F. J. Angerer P. Wolf F. A. (2021). Anndata: Annotated Data. bioRxiv.

[B57] Virtanen P. Gommers R. Oliphant T. E. Haberland M. Reddy T. Cournapeau D. (2020). SciPy 1.0: fundamental algorithms for scientific computing in python. Nat. Methods 17, 261–272. 10.1038/s41592-019-0686-2 32015543 PMC7056644

[B58] von Tell D. Armulik A. Betsholtz C. (2006). Pericytes and vascular stability. Exp. Cell Res. 312, 623–629. 10.1016/j.yexcr.2005.10.019 16303125

[B59] Wang X. Spandidos A. Wang H. Seed B. (2012). PrimerBank: a PCR primer database for quantitative gene expression analysis, 2012 update. Nucleic Acids Res. 40, D1144–D1149. 10.1093/nar/gkr1013 22086960 PMC3245149

[B60] Wicaksono S. A. Ryanto G. R. T. Suzuki Y. Hara T. Ikeda K. Fukumoto T. (2025). Endothelial senescence drives intrinsic skin aging via the neuroimmune CGRP-Mast cell axis in mice. Commun. Biol. 8, 1696. 10.1038/s42003-025-09097-2 41299075 PMC12658021

[B61] Wickham H. (2016). ggplot2: Elegant Graphics for Data Analysis. New York: Springer.

[B62] Wolf F. A. Angerer P. Theis F. J. (2018). SCANPY: large-scale single-cell gene expression data analysis. Genome Biol. 19, 15. 10.1186/s13059-017-1382-0 29409532 PMC5802054

[B63] Yu G. Wang L. G. Han Y. He Q. Y. (2012). clusterProfiler: an R package for comparing biological themes among gene clusters. OMICS A J. Integr. Biol. 16, 284–287. 10.1089/omi.2011.0118 22455463 PMC3339379

[B64] Yu Y. Ren Y. Li Z. Li Y. Li Y. Zhang Y. (2023). Myo1b promotes premature endothelial senescence and dysfunction via suppressing autophagy: implications for vascular aging. Oxid. Med. Cell Longev. 19:4654083. 10.1155/2023/4654083 36654782 PMC9842418

[B65] Zhan Y. Brown C. Maynard E. Anshelevich A. Ni W. Ho I. C. (2005). Ets-1 is a critical regulator of ang II-mediated vascular inflammation and remodeling. J. Clin. Invest 115, 2508–2516. 10.1172/JCI24403 16138193 PMC1193876

[B66] Zhang L. Pitcher L. E. Prahalad V. Niedernhofer L. J. Robbins P. D. (2023). Targeting cellular senescence with senotherapeutics: senolytics and senomorphics. FEBS J. 290, 1362–1383. 10.1111/febs.16350 35015337

[B67] Zou Z. Long X. Zhao Q. Zheng Y. Song M. Ma S. (2021). A single-cell transcriptomic atlas of human skin aging. Dev. Cell 56, 383–397.e8. 33238152 10.1016/j.devcel.2020.11.002

